# Towards two decades of Atlantic Ocean mass and heat transports at 26.5° N

**DOI:** 10.1098/rsta.2022.0188

**Published:** 2023-12-11

**Authors:** William E. Johns, Shane Elipot, David A. Smeed, Ben Moat, Brian King, Denis L. Volkov, Ryan H. Smith

**Affiliations:** ^1^ Rosenstiel School of Marine, Atmospheric and Earth Science, University of Miami, Miami, FL, USA; ^2^ National Oceanography Centre, Southampton, UK; ^3^ Cooperative Institute for Marine and Atmospheric Studies, University of Miami, Miami, FL, USA; ^4^ NOAA Atlantic Oceanographic and Meteorological Laboratory, Miami, FL, USA

**Keywords:** Atlantic meridional overturning circulation, ocean heat transport, ocean circulation time series, climate variability

## Abstract

Continuous measurements of the Atlantic meridional overturning circulation (AMOC) and meridional ocean heat transport at 26.5° N began in April 2004 and are currently available through December 2020. Approximately 90% of the total meridional heat transport (MHT) at 26.5° N is carried by the zonally averaged overturning circulation, and an even larger fraction of the heat transport variability (approx. 95%) is explained by the variability of the zonally averaged overturning. A physically based separation of the heat transport into large-scale AMOC, gyre and shallow wind-driven overturning components remains challenging and requires new investigations and approaches. We review the major interannual changes in the AMOC and MHT that have occurred over the nearly two decades of available observations and their documented impacts on North Atlantic heat content. Changes in the flow-weighted temperature of the Florida Current (Gulf Stream) over the past two decades are now taken into account in the estimates of MHT, and have led to an increased heat transport relative to the AMOC strength in recent years. Estimates of the MHT at 26.5° N from coupled models and various surface flux datasets still tend to show low biases relative to the observations, but indirect estimates based on residual methods (top of atmosphere net radiative flux minus atmospheric energy divergence) have shown recent promise in reproducing the heat transport and its interannual variability.

This article is part of a discussion meeting issue ‘Atlantic overturning: new observations and challenges’.

## 1. Introduction

The Atlantic meridional overturning circulation (AMOC) plays an important role in the global climate system through its meridional transport of heat, freshwater, carbon and nutrients. At 26.5° N, the Atlantic Ocean circulation carries about 1.2 PW (1 PW = 10^15^ W) of heat northward, which is approximately 60% of the net poleward heat flux carried by the global oceans and 30% of the total flux by the ocean and the atmosphere at this latitude [1,2]. This poleward heat flux is dominated by the AMOC, in which upper ocean waters moving northward in the basin are transformed into North Atlantic Deep Water and transported southward within the Deep Western Boundary Current (DWBC) and ocean interior [3–5].

Bjerknes [6] first hypothesized that variations of the Atlantic thermohaline circulation on decadal time scales are coupled to climatic variations. Coupled climate models have provided evidence that stronger AMOC states are linked to warmer temperatures in the North Atlantic, generally referred to as Atlantic multidecadal variability (AMV) [7–9]. The AMV produces widespread climate impacts, including global and regional precipitation anomalies (especially over the African Sahel, India and Brazil), summer climate over Europe and North America, and Atlantic hurricane activity [10–13]. The AMOC has also been linked to changes in Arctic surface air temperature and sea ice cover [14], and models indicate that large AMOC changes would lead to significant regional sea-level changes around the North Atlantic [15]. There is currently an ongoing debate about the role of the AMOC in driving the AMV, relative to the effects of natural (and human) aerosol forcing [16], and long-term observations of the AMOC are needed to determine its specific role in forcing the AMV and its related climatic impacts.

Attempts to understand the mechanisms of AMOC variability have suggested that atmospheric forcing associated with the North Atlantic oscillation (NAO) is a prime driver of AMOC variability, through both wind stress and surface buoyancy changes [17–21]. During positive phases of the NAO, westerlies are stronger over the North Atlantic and act to cool the surface of the subpolar North Atlantic Ocean through increased turbulent fluxes. Convection in the Labrador Sea generally increases during periods of high NAO, while it decreases in the Greenland Sea [22,23].

Model studies indicate that NAO-related variations in the heat fluxes over the subpolar North Atlantic induce a 2–3-year lagged response of the AMOC at mid-latitudes [9,17,19,20,24,25]. These NAO buoyancy forcing changes lead to meridionally coherent AMOC fluctuations throughout the North Atlantic, while wind stress forcing causes more localized and higher frequency responses (e.g. [19]). Changes in wind stress immediately affect the AMOC through variations in Ekman transports, which are balanced by barotropic transports on short time scales [21,26,27]. On longer time scales, the AMOC responds to wind stress curl changes through adjustment of the baroclinic pressure gradient across the basin by boundary processes and planetary waves [17,28–32]. The magnitude of wind and buoyancy-forced AMOC variations is believed to be of the same order, about 3–4 Sv (Sverdup, 1 Sv = 10^6^ m^3^ s^−1^) or roughly 15–20% of the mean strength of the AMOC [19,33].

**Figure 1.**
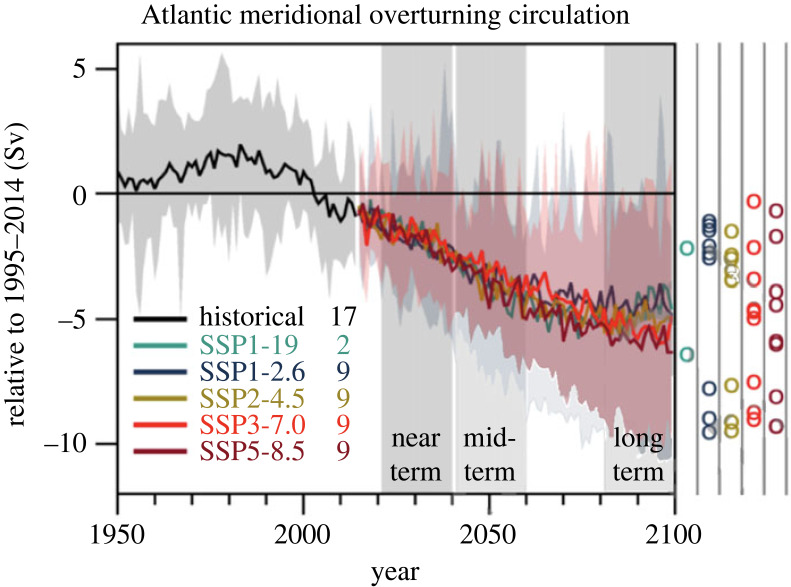
Adapted from IPCC AR6 WG1: CMIP6 annual mean AMOC strength change at 26° N in historical and scenario simulations. Changes are relative to averages from 1995 to 2014. The curves show ensemble averages and the shadings the 5–95% ranges across the SSP1–2.6 and SSP3–7.0 ensembles. The circles to the right of the panel show the anomalies averaged from 2081 to 2100 for each of the available model simulations. The numbers inside the panel are the number of model simulations.

The role of the AMOC in anthropogenic climate change scenarios is presently at the forefront of scientific debate. Since the release of the IPCC Assessment Report 5 (AR5, [34]) and the Special Report on the Ocean and the Cryosphere in a Changing Climate (SROCC; [35]), the newly released AR6 report has revised downward to ‘low' the confidence in modelled and reconstructed AMOC over the twentieth century due to new observations and model disagreement [36]. It is projected that the AMOC will decline in the twenty-first century but the models exhibit different magnitudes and timings (figure 1). The projected AMOC decline by 2100 ranges from 24% [4% to 46%] in scenario SSP1–2.6 to 39% [17% to 55%] in scenario SSP5–8.5 (with medium confidence).

Changes in Northern Hemisphere atmospheric climate, particularly over the Atlantic sector, depend crucially on how the AMOC will actually change under greenhouse forcing scenarios. Because the North Atlantic Ocean accounts for about 40% of the global CO_2_ oceanic uptake as a consequence of the AMOC, a reduced state of the AMOC would lead to reduced global oceanic CO_2_ uptake, reinforcing atmospheric CO_2_ concentration and climate change as part of a positive feedback loop [37–40].

While much progress has been made in recent years in modelling the AMOC and attempting to understand its variability mechanisms and potential impacts, a vital need within the climate modelling community is a long, observationally based time series of the AMOC that can be used to understand its linkages to surface forcing and to test model predictions. The RAPID programme has been making continuous measurements of the AMOC and associated ocean heat transport at 26.5° N for currently over 19 years, and is the only decadal-plus length observing system that provides full-depth estimates of the AMOC variability and structure across the basin. Other programmes that are currently measuring components of the basin-wide AMOC include the MOVE array at 16° N since 2000 (which measures the deep limb of the AMOC within the western basin, [41,42]), the SAMOC/SAMBA array at 34.5° S that was fully implemented in 2014 [43] and the OSNAP array across the subpolar North Atlantic that began in 2014 [44].

In this paper, we provide updated estimates of the AMOC strength and ocean meridional heat transport (MHT) across 26.5° N for the period from April 2004 to December 2020, which constitutes the latest update of the full time series from the data that have been recovered and processed to date. The methods for estimating the AMOC and MHT from the 26.5° N array are briefly reviewed, including new estimates of interannual variability of the heat transport carried by the Florida Current (Gulf Stream) and its impact on the overall basin-wide MHT variability. The breakdown of the total MHT into ‘gyre’ and ‘overturning' components is also discussed, where a comparison is made between the classical decomposition into a zonally averaged vertical overturning cell and a residual horizontal cell versus more heuristically based estimates. It is shown that the magnitude of the gyre component of the MHT can have widely different values depending on the assumptions made, leading to questions about the physical interpretation of the overturning and gyre components derived from Eulerian-based observations. The MHT estimates from the 26.5° N array are compared with estimates from available surface heat flux climatologies, showing that most surface heat flux datasets still substantially underestimate the true ocean heat transport. Finally, we review the documented impacts of the observed MHT variability at 26.5° on the interannual variability of ocean heat content in the North Atlantic over the period of the RAPID observations.

## 2. Data and methods

### (a) Estimates of the meridional overturning circulation

The AMOC observing system along 26.5° N is a collaborative effort among three independently supported research programmes: the U.K. Rapid Climate Change (RAPID), the US Meridional Overturning Circulation and Heat-flux Array (MOCHA) and the US NOAA Western Boundary Time Series (WBTS)program. We will refer to these programmes collectively as RAPID for short.

**Figure 2.**
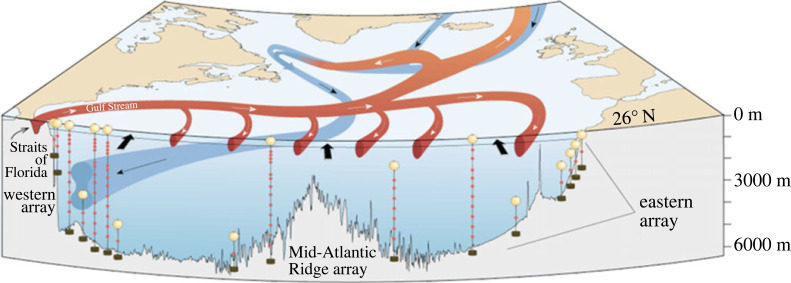
Schematic of the RAPID-MOCHA array and the main circulation components it measures: the Gulf Stream in the Straits of Florida, the upper mid-ocean gyre return flow, the deep interior flow including the Deep Western Boundary Current and the surface Ekman transport (indicated by black arrows).

The key components of the observing system are (figure 2):

(1) Gulf Stream transport through the Florida Straits measured by subsea electromagnetic cable, regularly calibrated by hydrographic and absolute velocity sections across the current [45,46];
(2) Ekman transport calculated from reanalysis wind stress, currently using ERA5 winds [47]; and
(3) Mid-ocean transport from the Bahamas to Africa measured by arrays of moorings at the eastern and western boundaries and on the flanks of the mid-Atlantic ridge.

**Figure 3.**
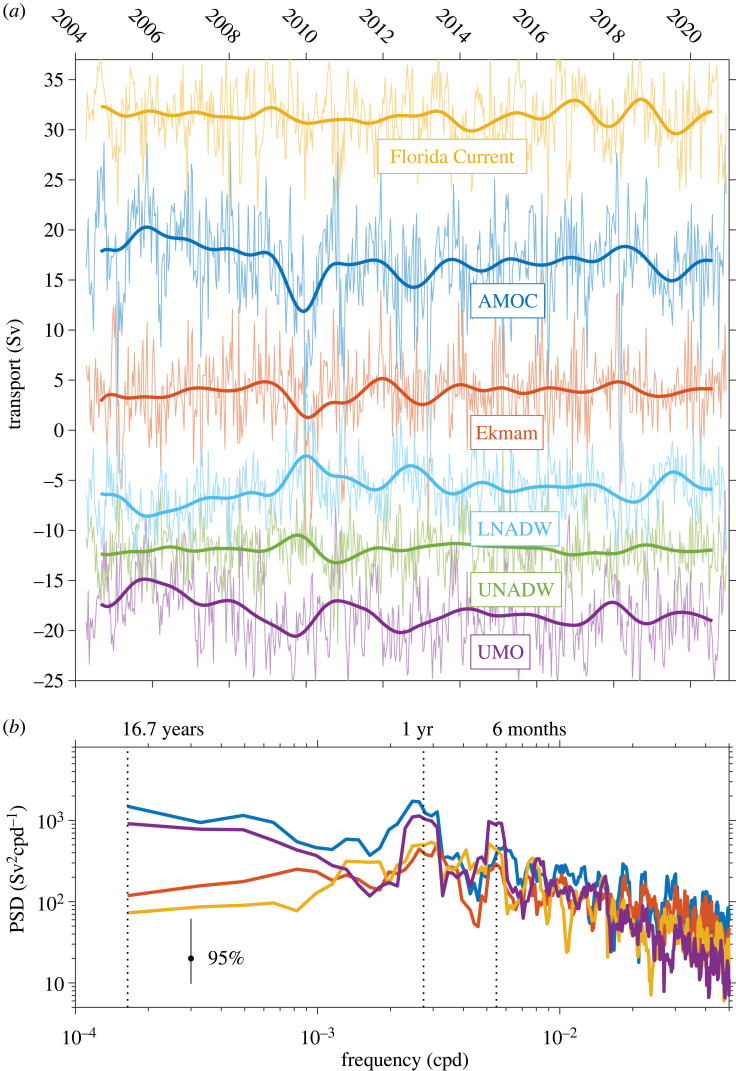
(*a*) Time series of Florida Current transport (yellow), Ekman transport (red), upper mid-ocean transport (purple) and overturning transport (dark blue) for the available record from 2004 to 2020. Also shown are the basin-wide transports in the upper and lower NADW layers (UNADW: 1100–3000 m; LNADW: 3000–5000 m; green and light blue lines, respectively). The high-frequency data represent 10-day averages while the superimposed curves are 18-month low-pass filtered data after removal of each series' climatological seasonal cycle. (*b*) Multi-taper spectra of the overturning circulation and its three upper ocean components (Florida Current, Ekman and UMO), following the same colour code as in (*a*).

The observational strategy relies on measurement of endpoint density profiles on either side of the basin to determine the interior basin-wide geostrophic shear, combined with direct observations of transport in the Antilles and DWBC region immediately offshore of the Bahamas (which we refer to hereafter as the ‘western boundary wedge'), and the transport though the Straits of Florida. Ekman transports are then combined with the geostrophic and direct current observations and an overall mass conservation constraint to continuously estimate the basin-wide AMOC strength and vertical structure [48–50]. The final result from the observing system is a vertical profile of the basin-wide integrated mass transport that, when vertically integrated, yields a profile of the AMOC streamfunction. The AMOC strength at any given time is defined as the maximum value of the streamfunction, which typically occurs near 1100 m, separating the upper warm northward flow of the AMOC from the lower cold southward flow of North Atlantic Deep Water (NADW). The upper flow can itself be divided into three components: the Ekman transport, the Florida Current transport and the part of the mid-ocean flow that lies above the AMOC maximum (which we refer to as the upper mid-ocean transport, UMO). These three components, along with the overall AMOC intensity, and the transports of the Upper and Lower NADW layers that make up the lower limb of the AMOC, are shown in figure 3 for the time series available to date.

### (b) Estimates of the meridional heat transport

The MHT carried across a trans-basin section at any latitude is given in [51]

$$Q=\int_{x_{w}}^{x_{e}}\int_{H}^{0}\rho c_{p}v\theta \;\text{d}x\;\text{d}z,$$

where *ρ* is seawater density, *c_p_* is the specific heat of seawater, *v* is meridional velocity, *θ* is potential temperature, and where the double integral is taken over the full depth (*H*) of the trans-basin section between eastern (*x_e_*) and western (*x_w_*) boundaries. Johns *et al.* [52] provided initial estimates of the MHT across 26.5° N by breaking this total heat transport down into a number of separate components of temperature transport (relative to a common temperature reference, typically 0°C), which are then summed together to derive the total MHT. The breakdown of terms used (as in [52]) is

2.1 $$Q_{\text{NET}}=Q_{\text{FC}}+Q_{\text{EK}}+Q_{\text{WBW}}+Q_{\text{MO}}+Q_{\text{EDDY}},$$

where the different terms represent, respectively, the meridional temperature transports of the Florida current (*Q*_FC_), the Ekman layer (*Q*_EK_), the western boundary wedge (*Q*_WBW_), the zonally averaged contribution by the mid-ocean circulation (*Q*_MO_) and the mid-ocean ‘eddy' contribution due to spatially correlated *v* and *θ* fluctuations (*Q*_EDDY_).

The methodology by which each of these terms is estimated is described thoroughly in Johns *et al.* [52], and we will only briefly review these here, with relevant updates. All terms that are dependent on temperature reference use a common 0°C temperature reference, and all references to temperature herein refer to potential temperature. The terms are estimated as follows:

#### (i) *Q*_FC_

The temperature transport of the Florida Current is obtained by multiplying the instantaneous cable-derived volume transport of the Florida current at 27° N by an estimate of the seasonally and interannually varying flow-weighted temperature (*θ*_FW_) of the Florida current. Since no continuous temperature measurements are available in the Florida current, the *θ*_FW_ of the Florida current is derived from the full ensemble of high-resolution shipboard sections of absolute velocity and temperature across the Florida current at 27° N that have been acquired during the time frame of the RAPID programme (totalling 101 sections from 2001–present), which are also used to validate the cable-derived volume transport. First, a seasonal climatology of *θ*_FW_ of the Florida current, defined as

**Figure 4.**
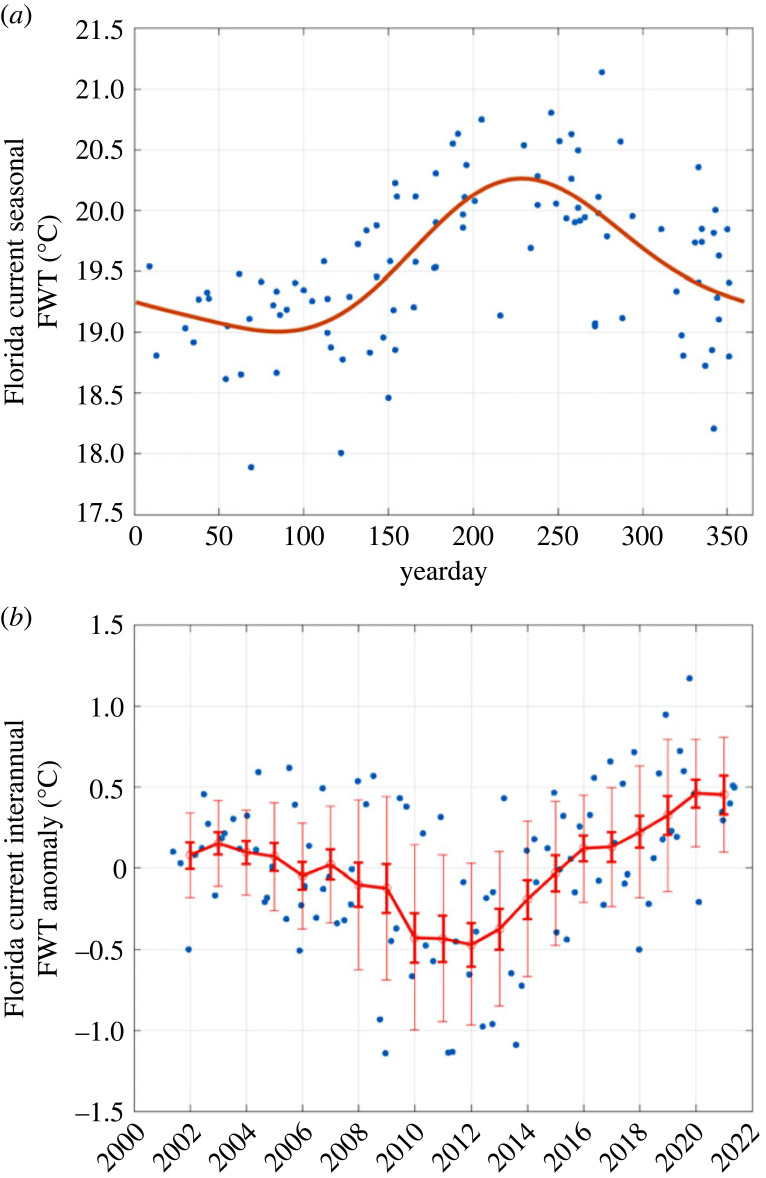
(*a*) Flow-weighted (potential) temperature of the Florida Current at 27° N (*θ*_FW_) as a function of yearday, from individual cross-sections occupied during 2001–2021 (blue dots), and the least-squares fit climatological seasonal cycle (sum of annual and semiannual harmonics; red line) derived from the individual section estimates. (*b*) Interannual anomalies of *θ*_FW_ with respect to the climatological seasonal cycle, taken as 3-year running means of the *θ*_FW_ section anomalies, centred on 1 January of each year. Thin error bars represent the standard deviation of all individual section anomalies over each 3-year averaging period; bold error bars represent the standard error of the section anomalies, assuming each section occupation is independent.

2.2 $$\theta_{\text{FW}}=\frac{\int v\theta \;\text{d}A}{\int v\;\text{d}A},$$

is estimated from the available section data, where *A* is the cross-sectional area of the Florida current and where a two-harmonic (annual plus semiannual) fit is used to obtain the climatological seasonal cycle, following Shoosmith *et al.* [53]. Then, interannual anomalies of *θ*_FW_ with respect to this best-fit seasonal cycle are estimated by a running 3-year average of all individual section estimates within a centred 3-year period around the date of the desired *θ*_FW_ estimate. The 3-year running mean of the individual section anomalies of *θ*_FW_ is somewhat subjective but is chosen to isolate the significant interannual variations of the *θ*_FW_ anomalies while also suppressing the relatively high non-seasonal variations of the individual section estimates (figure 4).

#### (ii) *Q*_EK_

The Ekman temperature transport is calculated using ERA5 wind stresses and the interior ocean temperature profiles derived from Argo (see below), where the Ekman layer temperature is taken as the average temperature of the upper 50 m of the water column.

#### (iii) *Q*_WBW_

The temperature transport in the ‘western boundary wedge' (WBW) region off the Bahamas, shoreward of the western endpoint dynamic height mooring used for the interior geostrophic calculation, is calculated from direct current and temperature measurements on the moorings across this region, as described in Johns *et al.* [52].

#### (iv) *Q*_MO_

This term represents the contribution to meridional temperature transport across the mid-ocean region by the zonally averaged flow and zonally averaged temperature on constant depth surfaces

2.3 $$Q_{\text{MO}}=\int \rho c_{p}\langle V\rangle \langle \theta \rangle \;\text{d}z,$$

where ⟨V⟩ is the zonally averaged transport profile from the mid-ocean AMOC calculation and ⟨θ⟩ is the zonally averaged temperature across the mid-ocean region derived from an objective analysis (OA) of available Argo data profiles combined with T/S profiles from the RAPID moorings. This OA product is produced by the RAPID programme at weekly temporal resolution, and merged into a seasonal climatology for the deep ocean interior (greater than 2000 m) based on EN4 data.

#### (v) *Q*_EDDY_

This term represents the contribution to meridional temperature transport by spatially correlated *v* and *θ* fluctuations across the mid-ocean region, given by

2.4 $$Q_{\text{EDDY}}=\int \int \rho \;c_{p}\;v^{\prime }\theta^{\prime }\text{d}x\;\text{d}z,$$

where *v*^′^ are geostrophic velocity anomalies relative to the zonal mean derived from the Argo/RAPID OA using a geostrophic approximation referenced to 1000 m, and *θ′* are the respective temperature anomalies with respect to the zonal mean. The estimates of *Q*_EDDY_ thus obtained typically range between 0 to 0.13 PW and are consistent with the range of estimates available from trans-basin shipboard hydrographic sections along 26.5° N [52]. As noted in Johns *et al.* [52], this ‘eddy' heat flux is actually associated mainly with the large-scale structure of *v* and *θ* anomalies across the subtropical gyre, rather than mesoscale features. The Argo data are therefore able to resolve it adequately even at relatively coarse resolution across the section.

## 3. Results

### (a) AMOC variability at 26.5° N

The AMOC shows large short-term variability at 26.5° N with 10-day estimates varying over a range of about 0–30 Sv (figure 3*a*). The Gulf Stream, Ekman and UMO transports all contribute to the AMOC variability, but their respective contributions are a strong function of time scale, with the UMO having dominated the low-frequency variability thus far (figure 3*b*).

The first 4 years of the AMOC time series were dominated by an annual cycle, which was shown to be caused by seasonal wind stress curl variations mainly in the eastern part of the basin, and that can be largely explained by wind-forced Rossby wave dynamics [30,31,54]. In 2009, the observed seasonal cycle began to break down and instead a large negative AMOC anomaly occurred in the winter of 2009–2010 with sustained values below 10 Sv. This low AMOC state was forced in part by reduced Ekman transports associated with extreme low values of the NAO index in winter 2009–2010, which recurred again in winter 2010–2011. However, the geostrophic mid-ocean circulation (UMO) also increased in strength (trending towards more southward transport) after 2008 leading up to this event, which also contributed to its intensity [55]. The 0.6 Sv yr^−1^ decline of the AMOC over the period from 2007 to 2011 was almost 10 times larger than the rate of long-term decline predicted by the IPCC as a result of climate change [56], and is believed to represent an interannual anomaly linked to either wind or buoyancy forcing over the North Atlantic rather than an anthropogenically forced change. Most of the interannual AMOC variability over this period can be reproduced by relatively simple wind-forced models [32,57], suggesting that the 2009–2010 event may have been primarily a wind-forced response.

However, since 2011 the AMOC has continued to occupy a weaker state relative to the first years of observations, with annual mean values in the range of 15–17 versus 18–19 Sv during 2004–2008. It is not clear that this can be explained by wind forcing and it has been suggested that the overall downtrend of the AMOC over the RAPID time period is linked to reduced convection in the Labrador Sea after 2000 that propagates via density anomalies to the subtropics on time scales of 7–8 years [20,58]. Over the 2005–2011 period, we observed a decreasing trend in densities within the DWBC off Abaco (not only within the Labrador Sea Water layer but through the whole NADW water column from approximately 1200–4500 m; [42]), which could be consistent with this explanation. Some part of this downtrend could also be linked to anthropogenically forced changes, but the observational record is still too short to discern a forced trend from decadal scale internal variability.

A remarkable aspect of the observed reduction in the AMOC is that the deep flows within the lower limb of the overturning cell that balance the upper ocean AMOC changes are concentrated mainly in the lower NADW (LNADW) layer between 3000 and 5000 m (figure 3). This is a direct result of the decreasing density through the entire NADW layer observed at the western side of the basin: the deepening of isopycnals at the western boundary causes an increased tilt of the deep isopycnals across the basin and greater northward shear below the mid-depth maximum of the southward NADW flow (near 2000 m), resulting in a reduced basin-wide LNADW flow. We find that this behaviour also occurs on shorter time scales associated with the abrupt AMOC reductions during winter 2009–2010 and 2010–2011 (where the basin-wide LNADW flow reduced to almost zero), and again in 2013, where in each case there is an abrupt reduction in the LNADW flow and an enhanced shear between the upper NADW (UNADW) and LNADW layers, with no clearly apparent impact on the UNADW layer. These short-term changes in the LNADW flow appear to be clearly linked to changes in the Ekman transport (figure 3*a*), but the observed behaviour is at odds with theoretical and modelling results that suggest that the mid-ocean compensation for Ekman transport changes should be primarily barotropic on these time scales [26,27]. The dynamics of these variations are still not fully understood and are an ongoing focus of investigation within the programme.

The overall results from the programme therefore show a decadal time-scale reduction of the AMOC—superimposed on large-amplitude interannual variability—that is characterized by an increased southward flow in the upper part of the mid-ocean circulation and a weakening of the southward transport of LNADW. While much work remains to be done in attributing these changes to forcing mechanisms, the RAPID time-series continues to provide essential long-term data for testing hypotheses and model results on their causes, through not only the AMOC time series at 26.5° N but the associated measurements of the western boundary current (WBC) system (Florida and Antilles Currents), interior flows and deep density variations across the basin.

The average strength of the AMOC over the entire period of the RAPID array to date (April 2004–December 2020) is 16.9 ± 1.2 Sv (table 1), where the uncertainty estimate includes both statistical uncertainties based on the variance of the time series as well as possible bias errors, following the methodology of Johns *et al.* [52]. The time mean AMOC strength at 26.5° N is remarkably consistent with estimates from other latitudes in the Atlantic, which range from 16.7 to 17.3 Sv from arrays in the subtropical South Atlantic to the North Atlantic subpolar gyre (table 1). Thus, despite the different time periods covered by the available array observations, the AMOC strength appears to be nearly uniform throughout the Atlantic basin. This result is also consistent with most available model results that show a nearly constant AMOC strength throughout the basin [62,63], when computed in either depth or density coordinates as appropriate for the latitude in question [44,63].

**Table 1.** The time mean AMOC strength at the SAMBA (34.5° S), MOVE (16° N), RAPID (26.5° N) and OSNAP (approx. 59° N) arrays from the latest available estimates at each location. Uncertainty estimates for the RAPID array include both statistical uncertainty based on the variance of the time series as well as possible bias errors, following Johns *et al.* [52]; the uncertainties of the other estimates are taken from the cited papers. At the SAMBA array (*), the time mean AMOC estimate uses a model-based interior reference velocity, while at the OSNAP array (†) the AMOC strength is computed in density coordinates.

latitude	array name (reference)	time period	AMOC strength (Sv)
34.5° S	SAMBA (Kersalé *et al.* [59])	2013–2017	17.3 ± 5.0*
16° N	MOVE (Volkov *et al.* [60])	2000–2019	17.3 ± 1.4
26.5° N	RAPID (this study)	2004–2020	16.9 ± 1.2
59° N	OSNAP (Fu *et al.* [61])	2014–2020	16.7 ± 0.6†

### (b) Interannual variability of ocean meridional heat transport

While the time-mean AMOC strength appears to vary only weakly with latitude through the Atlantic, the ocean MHT varies dramatically. Available estimates from trans-basin hydrographic sections and from the AMOC arrays that currently produce MHT estimates show an increase from approximately 0.5 PW in the subtropical South Atlantic to a maximum value of near 1.2 PW in the tropical and subtropical North Atlantic, declining again northward to values of approximately 0.5 PW at 60° N and to approximately 0.3 PW at the northern terminus of the basin where waters in the upper limb of the AMOC enter the Nordic Seas [1,43,64–66]. The large increase in the MHT from the subtropical South Atlantic to the subtropical North Atlantic occurs mainly through interaction of the large-scale thermohaline AMOC cell with the shallow wind-driven overturning cells in the tropics—the so-called ‘subtropical cells' [67,68]—which result in an uplift and warming of thermocline waters as they cross the equator into the North Atlantic within the upper limb of the AMOC. By this process, and the associated strong diapycnal conversion of waters due to upwelling near the equator, the thermocline waters that comprise the bulk of the upper AMOC limb in the South Atlantic are converted to much warmer surface waters that subsequently continue northward in the upper limb of the AMOC, both within the WBC system (Gulf Stream) and in the surface Ekman layer [68]. Farther northward, the strong surface heat loss to the atmosphere in mid-latitudes and over the subpolar gyre causes a progressive cooling of the upper limb waters and a reduction of their temperature difference with respect to the cold waters of the deep limb, resulting in a gradually reduced heat transport. Effectively, it is the change in the flow-weighted temperature of the upper limb as it progresses northward—in the context of a nearly uniform AMOC strength—that determines the magnitude of the MHT. The MHT at 26.5° N, which is near the latitude of maximum heat transport, is of great importance because its variability determines the amount of heat that is delivered to the rest of the North Atlantic, which can feed back on air–sea interaction processes and ultimately impact processes such as deep water formation.

As described in §2, the MHT at 26.5° N is estimated by combining several measured components of temperature transport (relative to a common temperature reference, here 0°C). The dominant northward component of temperature transport is in the Florida Current (*Q*_FC_), and here we introduce updated estimates of *Q*_FC_ that include, for the first time, estimates of the interannually varying flow-weighted temperature (*θ*_FW_) of the Florida Current. Previously, the estimates of *Q*_FC_ had only included the climatological seasonal variability of *θ*_FW_ [50,52,69]. The estimates of *θ*_FW_ from all of the available high-resolution sections across the Florida Current during the RAPID period (figure 4*a*) show a seasonal cycle that is very similar to that of Shoosmith *et al.* [53], and to periodic updates that have been made throughout the RAPID programme using newly available section data. The average value of *θ*_FW_ is 19.6°C, and the range of the seasonal cycle is 1.2°C with a maximum in mid-August (yearday 230) and a minimum in mid-March (yearday 80). There is considerable variability within any given month, and the uncertainty about the best-fit annual cycle is approximately ±0.2°C.

The interannual anomalies of *θ*_FW_ with respect to this climatological seasonal cycle, estimated as described in §2, also show a considerable amount of random variability (figure 4*b*), but clearly indicate a drop in 2010–2013 and a relatively steady rise since that time to the highest values in 2020–2021, the most current section measurements available. The range of variability of the interannual signal is approximately 1°C, the same order as that of the seasonal cycle. Up until the last several years of section data, it was unclear if the yearly anomalies were significant within the spread of the individual section *θ*_FW_ estimates, but the interannual signal now seems sufficiently robust that it should be included in the time-series estimates of *Q*_FC_.

**Figure 5.**
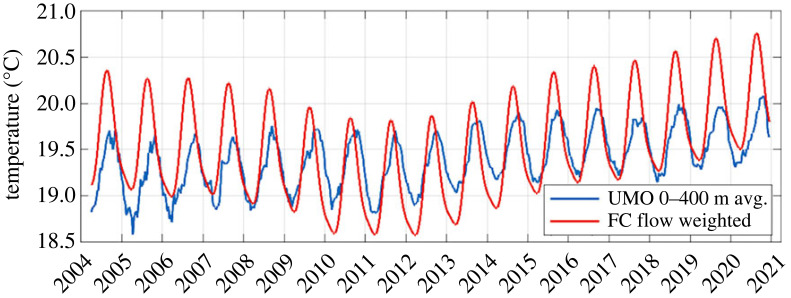
Seasonal plus interannual variation of the Florida Current flow-weighted temperature (red) versus the ocean interior temperature averaged over the upper 400 m (blue).

When combined, the seasonal plus interannual variability of *θ*_FW_ is as shown in figure 5, where it is compared with the average 0–400 m temperature across the ocean interior where the largest part of the gyre flow is returned southward. Both time series show an increase since 2012, but the warming in the Florida Current is larger, leading to a tendency for increasing overall MHT. Furthermore, since only about half of the Florida Current transport is returned in the subtropical gyre (the mean Florida Current and combined Ekman/UMO transports are 31.4 and 14.3 Sv, respectively), the increasing temperature trend in the Florida Current will lead to a larger temperature difference between the portion of Florida Current waters that continue to move northward in the Atlantic and are ultimately returned as NADW, leading to a directly proportional increase in the heat transport by the large-scale overturning cell (since changes in overall NADW temperatures can be expected to be at least an order of magnitude smaller). The observed changes in *θ*_FW_ reflect changes in the overall mean temperature of the Florida Current over the past two decades, and are not linked in any intricate way to the flow-weighting of the temperature field. As shown by Domingues *et al.* [70], the warming since 2012 occurs all across the Straits, mainly within the upper 300 m, and is linked to changes in upper ocean heat content in the Gulf of Mexico, Caribbean Sea and tropical Atlantic regions upstream of the Florida Current. The change in the 3-year running mean of *θ*_FW_ from its low in 2012 to the end of the record in 2020–2021 was about 0.9°C, which corresponds to a 0.11 PW change in *Q*_FC_ (for a constant volume transport of 31.4 Sv) or about 4.5% of the record-mean value of *Q*_FC_ of 2.50 PW. This change in *θ*_FW_ also results in a difference of approximately 0.11 PW in the total MHT between those two periods, when compared against a calculation that uses only the climatological seasonal variation of *θ*_FW_ without the interannual anomaly.

**Figure 6.**
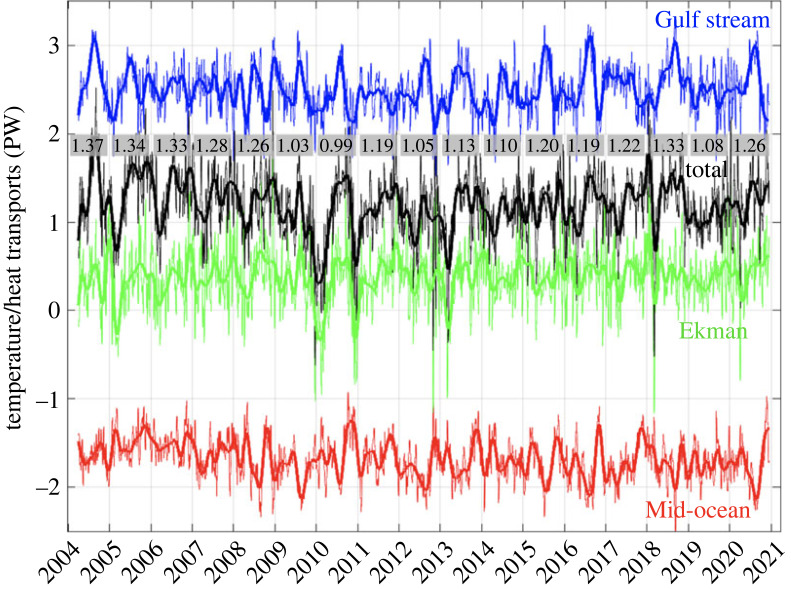
Time series of net meridional heat transport (black), and temperature transports (relative to 0°C) of the Gulf Stream (blue), Ekman layer (green) and Mid-ocean region (red). Thin lines are 10-day averages and thick lines are 90-day low-pass filtered results. The Mid-ocean temperature transport shown here is the sum of all three terms across the ocean interior between the Bahamas and Africa: *Q*_WBW_, *Q*_MO_ and *Q*_EDDY_. Annual averages of the net heat transport are shown in grey boxes.

The time series of the total MHT, and its associated temperature transport components in the Florida Current, Ekman layer and mid-ocean region are shown in figure 6, together with the annual averages of the total MHT. The annual averages for the first 5 years (2004–2008) were close to 1.3 PW, followed by a drop to values near 1.0 PW during 2009–2010, after which the MHT recovered to values of 1.05–1.22 PW during 2011–2017 but still consistently lower than in the first 5 years of measurement. Only in the last few years (2018 and 2020) have the annual mean values begun to approach the approximately 1.3 PW values that were observed in the first 5 years of measurement.

The time-mean MHT over the total duration of the measurements (2004–2020) is 1.20 ± 0.12 PW, where the uncertainty includes both the statistical error in the mean MHT (based on the variance of the time series and its decorrelation time scale, approx. 40 days) as well as potential measurement bias errors, following the methodology of Johns *et al.* [52]. Considering multi-year averages, the mean MHT from 2004 to 2008 was 1.32 ± 0.05 PW, while for 2009–2015 it was 1.10 ± 0.08 PW, a nearly 20% decrease. The error bars on the above represent the standard deviations of the yearly mean values from each period in order to provide a relative comparison of the MHT strength for different periods. Even when excluding the extreme low values of the MHT during 2009–2010, the mean MHT over the last decade of measurements (2011–2020) is 1.17 ± 0.09 PW, about 0.15 PW smaller than during the first 5 years of measurement. Such large changes should be expected to have a measurable impact on the heat budget of the North Atlantic on interannual to decadal time scales, and evidence for such impacts are discussed later in §4 of the paper.

**Figure 7.**
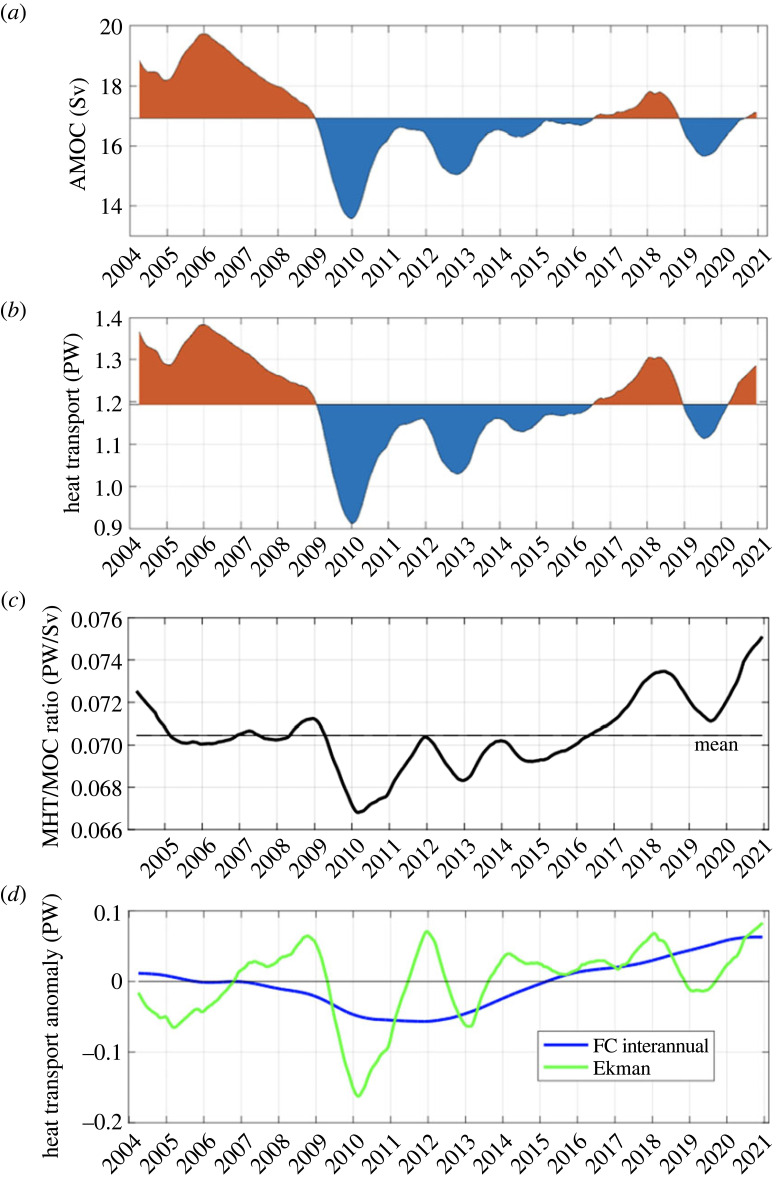
Running 1-year mean of the (*a*) AMOC and (*b*) MHT time series; anomalies with respect to the record-length means shown by red (positive) and blue (negative) shading. (*c*) Ratio of the MHT to the AMOC strength. (*d*) Main contributions to the interannual variability of the MHT/AMOC relationship, due to variability in the Ekman (green) and Florida Current (blue) contributions to the heat transport.

It is of interest to compare the relative strengths of the AMOC and MHT over the period of the RAPID programme. While the MHT continues to show a very high correlation with the AMOC strength (*r* = 0.96 for 10-day averages), their relationship has subtly changed over time, in particular when focusing on interannual time scales (figure 7). The drop in MHT during 2009–2010 was more pronounced than that of the AMOC, and the recovery to higher values since then is more apparent in the MHT time series, as shown by the higher positive anomalies from 2016 onward. The average ratio of the MHT to the AMOC (figure 7*c*) is approximately 0.7 PW/Sv, but it has varied over a range of about 10% of its mean value, with the highest ratios occurring in the last few years of observation. As shown in figure 7*d*, the interannual variations in the MHT/AMOC ratio are mostly related to changes in the Ekman heat transport, which has a larger relative impact on the MHT than on the AMOC due to the very warm temperatures of the Ekman layer. However, the longer-term variation of the MHT/AMOC ratio is mainly related to the changes in Florida Current heat transport, which are in turn linked to the variation in the flow-weighted temperature of the Florida Current that are now included in the overall MHT estimate.

### (c) Overturning and gyre heat transports

**Figure 8.**
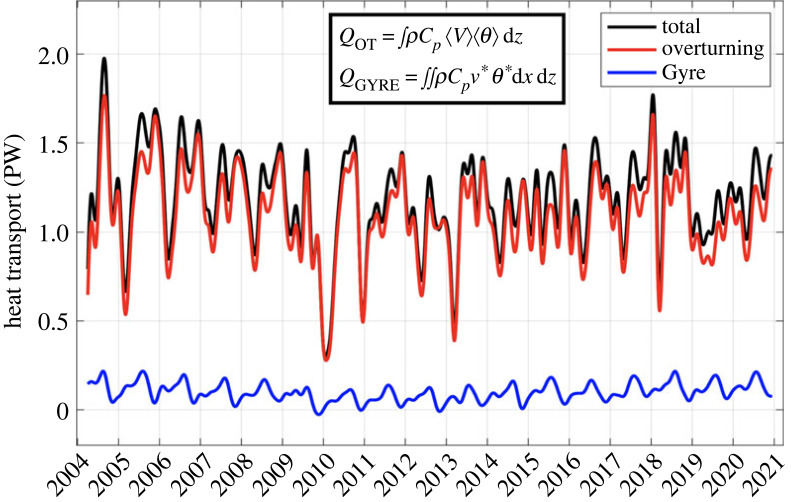
Heat transport decomposition into an ‘overturning' component (*Q*_OT_, derived from the zonally averaged velocity and temperature in depth coordinates, red curve), and a ‘gyre' component (*Q*_GYRE_, derived from velocity and temperature anomalies with respect to the zonal mean, also computed in depth coordinates, blue curve). Total heat transport is shown in black; all time series are 90-day low-pass filtered.

The total MHT can be decomposed into contributions by the zonally averaged flow and anomalies with respect to that zonally averaged flow, which are often referred to as the ‘overturning' and ‘gyre' heat transports, respectively [51,71]. The definitions of these contributions are the same as those used for the Q_MO_ and Q_EDDY_ breakdown of the interior temperature transport (equations (2.3) and (2.4), respectively), except that the zonally averaged quantities now represent averages across the full basin-wide section (including the Florida Current and WBW region), and the anomalies are also with respect to those full basin-wide zonal means [52]. The above breakdown into basin-wide vertical and horizontal cells (figure 8) shows that 92% (1.10 PW) of the total time-mean MHT at 26.5° is carried by the vertical overturning circulation and 8% (0.10 PW) is carried by the residual horizontal circulation. Furthermore, 95% of the total MHT variance is accounted for by the variance of the overturning component. The horizontal cell component has weak interannual variability and is mainly dominated by a seasonal cycle, which involves the seasonal cycles of the Florida Current and upper ocean interior flows but also reflects a difference in the amplitude and phase of the seasonal cycle of temperatures in the Florida Current and upper ocean interior (figure 5).

The above simple breakdown into vertical and horizontal cells has been criticized for not providing a meaningful measure of the actual ‘gyre' component of heat transport, because the waters flowing northward in the Gulf Stream that recirculate in the gyre do not, in general, return on constant depth surfaces (e.g. [72]). In fact, some portion of the Gulf stream waters that recirculate in the gyre (as well as all of the northward Ekman transport at 26.5°) is subducted into the thermocline by Ekman pumping and returns southward at greater depths (and densities) than the original Gulf Stream waters. Talley [72] made two estimates of the gyre component of heat transport by assuming that the Florida Current waters that recirculate southward in the gyre were either (a) uniformly distributed through the entire density range of the Florida Current above the maximum density of the interior southward flow, or (b) completely contained within the lightest (warmest) layers of the Florida Current. At 24° N, Talley [72] estimated these values to be (a) 0.09 PW and (b) 0.40 PW, obviously indicating a large range of possible estimates depending on the assumptions made.

We can perform a calculation similar to that of Talley's by using the average results of all the Florida Current sections over the RAPID period to estimate the mean transport of the Florida Current in density coordinates, combined with an estimate of the interior transport in density coordinates using the interior Argo data. The temperature transports in each density class are also calculated for both the Florida Current and ocean interior. We find that the interior southward gyre flow is confined above a density level of *σ_θ_* = 27.3, identical to that of Talley at 24° N (and also consistent with the maximum winter outcrop density in the North Atlantic at the zero wind stress curl along the northern edge of the subtropical gyre; [72]). The total southward geostrophic transport across the gyre is 20.4 Sv, which, when combined with the northward Ekman transport of 3.8 Sv, yields a net southward transport of 16.6 Sv that needs to be supplied by northward flow within the WBC system of the subtropical gyre. Different from Talley [72], we include the upper ocean transport in the western boundary region off the Bahamas, where a northward transport occurs in the Antilles current, together with the Florida Current in the estimates of the total WBC transport. The corresponding estimates of the gyre heat transport by balancing this 16.6 Sv of flow by northward transport in the WBC system, using the same assumptions (a) and (b) as in Talley [72], are 0.11 and 0.43 PW, respectively, very similar to those of Talley [72].

This large range of possible estimates raises the question of what waters in the WBC system are physically recirculated within the subtropical gyre. Based on observed surface drifter trajectories and model-based float tracking studies, there is good reason to believe that a large portion of the waters that recirculate in the gyre come from the very near-surface layers of the Florida Current. Brambilla & Talley [73] found that only one of 273 surface drifters launched in the Gulf Stream east of Cape Hatteras left the subtropical gyre to move into the subpolar gyre. Burkholder & Lozier [74] similarly found that very few (less than 2.5%) of the surface drifters launched in the Gulf Stream in their model study reached the subpolar gyre. They further found that less than 15% of synthetic floats launched in the upper 200 m of the Gulf Stream and tracked along three-dimensional pathways reached the subpolar gyre. The implication of these studies is that most of the near-surface waters in the Gulf Stream are recirculated in the subtropical gyre, which are then cooled and subducted to form the denser waters that flow southward in the gyre interior. The transport in the upper 200 m of the Florida Current from the average of all the Florida Current sections is 16.7 Sv, very close to the required 16.6 Sv of recirculating flow from the WBC system. Therefore, if most of the waters recirculating in the gyre are drawn from this upper 200 m of flow in the Florida Current, the resulting estimate of the gyre heat transport would be closer to the top end of Talley's (or our) estimates of approximately 0.4 PW.

Burkholder & Lozier [74] also showed that waters within the Gulf Stream that reached the subpolar gyre were preferentially drawn from intermediate and thermocline levels in the Gulf Stream. Burkholder & Lozier [75] later proposed a ‘surface to subsurface spiral' in the subtropical gyre in which surface Gulf Stream waters are converted to denser waters flowing southward in the gyre, which then supply the thermocline level flow in the Gulf Stream that subsequently moves northward into the subpolar gyre as part of the large-scale AMOC cell. Notably, water mass studies have shown that the lightest waters in the Florida Current (greater than 24°C, comprising approx. 11 Sv of the total FC transport during the RAPID period) are mostly derived from South Atlantic and tropical surface waters moving northward in the upper limb of the large-scale AMOC cell [76]. Therefore, in this scenario, to add to the conceptual model of Burkholder & Lozier [75], the gyre is, to a large degree, acting to modify surface flows within the upper limb of the large-scale interhemispheric AMOC into cooler and saltier thermocline waters that then continue northward again in the large-scale AMOC. The physical interconnectivity of the two circulations is one of the reasons that dividing the meridional water mass modification, and the net heat transport, into parts carried by either circulation is challenging (see [77]).

In addition to the classical decomposition of the MHT into horizontal and vertical cells in depth space, it is possible to perform an analogous calculation in density space where the relevant quantities (zonal mean velocities and temperatures, and their anomalies) are calculated along constant density surfaces rather than on constant depth surfaces. Xu *et al.* [78] performed this calculation at 26.5° N in a numerical model study and found an overturning (diapycnal) heat transport of 1.29 PW and a horizontal (isopycnal) heat transport of −0.05 PW, adding up to their total model heat transport of 1.24 PW. The same calculation performed on the RAPID data yields respective estimates of 1.32 and −0.12 PW for these quantities, adding up to the total heat transport of 1.20 PW. In both cases, the negative isopycnal contribution by the gyre circulation is because waters flowing northward in the Florida Current are colder (and correspondingly fresher) on the same density surfaces than the waters flowing southward in the gyre interior. This is well known from observations [76] and is also reproduced by the Xu *et al.* [78] model results, and can be explained by the fact that the high salinities of subtropical gyre thermocline waters are gradually eroded by mixing with fresher waters in the Caribbean Sea and Gulf of Mexico along their pathway into the WBC system. The lower salinities in the Caribbean Sea and Gulf of Mexico are, in turn, related to the influence of tropical waters moving northward in the upper branch of the large-scale AMOC via the tropical WBC system and by North Brazil Current rings [76,79].

Based on this negative isopycnal gyre contribution, and an offsetting small northward heat transport of 0.06 PW linked to subtropical mode water (STMW) formation, Xu *et al.* [78] postulated that the net heat transport by the gyre circulation was effectively zero. They argued that Talley's high-end estimate was too large because, in their words, ‘the near-surface layer of the Florida Current is dominated by relatively fresh water from the tropical/South Atlantic that is best regarded as part of the larger-scale AMOC'. However, this neglects the possibility that much of this surface water in the Florida Current is converted to thermocline waters as part of the surface to subsurface gyre loop and subsequently continues northward in the large-scale upper AMOC limb. Consequently, a significant part of the diapycnal overturning heat transport may actually be occurring in the gyre. Therefore, in our view, the breakdown of the heat transport into a net diapycnal overturning and an isopycnal horizontal contribution is not directly useful in diagnosing the effective heat transport carried by the gyre or large-scale overturning circulations.

The point of this discussion—absent any definite conclusion—is that dividing the total MHT into a part carried by the large-scale overturning circulation and a part carried by the gyre is far from trivial, and we submit that none of the above estimates can be taken as a reliable estimate of the gyre component of the MHT. To determine the gyre heat transport accurately requires knowledge of how (and which) water parcels flowing northward in the WBC system are returned to the gyre, and what their associated temperature changes are. We believe the breakdown of the MHT into gyre and overturning components is ultimately a Lagrangian problem, and will return to this topic in the discussion section where it is proposed as a challenge for the AMOC observing and modelling community.

## 4. Discussion

The AMOC and MHT estimates from the RAPID array have provided a valuable benchmark for assessment of numerical models and surface heat flux datasets. Historically, models have tended to underestimate the MHT at latitudes near 26° N in the Atlantic, even if producing realistic estimates of the AMOC strength. For example, Liu *et al.* [80] recently showed that nearly all AMIP6 models (Atmospheric Model Intercomparison Project Phase 6; [81]) underestimated the MHT at 26° N, with the ensemble mean of all models over the period from 1985 to 2015 showing a mean MHT about 0.3 PW lower than the 1.2 PW estimate from RAPID. A similar bias is found in coupled models, where a large ensemble of models from CMIP6 were shown to underestimate the MHT by about 0.2 PW relative to the RAPID MHT estimate [82]. Besides the actual magnitude of the MHT, the relative strengths of the MHT and AMOC can also be a valuable diagnostic in assessing models, since model biases can lead to an underestimate of the MHT even if the AMOC strength is approximately correct (e.g. [83]).

Most of the commonly used air–sea heat flux datasets (NOC, NCEP, ERA5, JRA-55, MERRA-2, CERES + OAFlux) also underestimate the MHT at 26° N, some of them quite considerably, due to too-weak surface heat losses over the North Atlantic [69,80]. Recent estimates using ‘residual' methods (i.e. top of atmosphere net radiative flux minus accumulated total column atmospheric energy divergence) have shown MHT estimates that are closer to the observed RAPID value. For example, Trenberth & Fasullo [2] find a mean MHT value of 1.04 PW at 26° N for the period from 2014 to 2016 (compared with a mean MHT of 1.18 from the overlapping RAPID period), while Liu *et al.* [80] find a mean MHT of 1.22 PW for the period from 2004 to 2017 (compared with 1.19 PW from RAPID over that same period). The residual methods also appear to more faithfully reproduce the temporal variability of the MHT observed during RAPID, after taking into account variations of the total ocean heat storage over the North Atlantic poleward of 26° N [2,69,80].

The observed changes in the AMOC and MHT during the RAPID period have been shown to have a pronounced impact on the ocean circulation and ocean heat content over the North Atlantic. Cunningham *et al.* [84] and Bryden *et al.* [85] showed that the large reduction in MHT during 2009–2010 caused a dramatic cooling of the northern subtropical gyre—the largest heat content drop observed over that region in 60 years—which had follow-on impacts on western European winter climate and the subsequent development of the NAO in winter of 2011 [86,87]. Volkov *et al.* [88] also showed that this event led to heat convergence in the tropical North Atlantic and produced wind-driven and thermosteric sea-level anomalies that affected sea levels along the eastern boundary as well as in the Mediterranean. There are also indications that the AMOC-modulated heat advection can affect the interannual-to-decadal changes of sea level along the US southeastern seaboard [70,89].

Considering the longer-term AMOC/MHT decline after 2009, Smeed *et al.* [90] showed that this change in AMOC state was concurrent with other changes in the North Atlantic, including a broadening of the Gulf Stream—evidenced by a change in the currents observed by satellite altimeter—and by altered patterns of ocean heat content and sea-surface temperature that resemble the pattern of response to a declining AMOC predicted by coupled climate models. More recently, Bryden *et al.* [69] have shown that the reduction in the AMOC between two reference periods in 2004–2009 and 2009–2016 resulted in a systematic cooling and freshening of the North Atlantic, due to the associated reductions in heat and salt transport across 26.5° N. While the extreme 2009–2010 AMOC event mainly impacted the northern subtropics, the sustained MHT reduction since 2009 appears to have had its greatest impact on the subpolar gyre, where broad cooling of up to 2°C in SST has occurred since 2009 [69].

The actual pattern of the maximum cooling and freshening—concentrated in the eastern subpolar gyre—was influenced by other factors, including advective changes in the subpolar gyre that led to a strong diversion of relatively cold/fresh upper ocean waters from the Labrador Current into the eastern subpolar gyre [91], as well as anomalously strong heat loss in the eastern subpolar North Atlantic during the winters of 2013–2015 [92,93]. Fox *et al.* [94] found that about two-thirds of the cooling and freshening over this region could be accounted for by anomalous advective flux from the Labrador current and one-third from reduced advection of warm salty waters from the subtropical gyre, which is consistent with the impacts of a reduced AMOC across 26° N. However, as shown by Bryden *et al.* [69], if it were not for the offsetting effects of reduced surface heat loss over the North Atlantic as a whole (poleward of 26° N) after 2009, the sustained drop in AMOC and MHT across 26° N would have been expected to lead to an overall cooling of the North Atlantic three to four times larger than observed.

The observed change in the MHT/AMOC relationship at RAPID in recent years (i.e. the increase in the MHT/AMOC ratio, figure 7) raises the question of how this relationship may be expected to change under global warming scenarios. It stands to reason that, as the upper layers of the ocean warm and the average temperatures of upper limb of the AMOC increase, the total heat transport by the large-scale Atlantic overturning cell (per unit AMOC) will increase. Although deep waters may also experience warming, their temperature changes would be expected to lag the upper ocean and to be at least an order of magnitude smaller, so that the average temperature difference between the upper and lower limbs of the AMOC would be expected to increase and lead to a proportionally larger heat transport. To give a rough order of magnitude of the effect, the average temperature difference between the upper and lower limbs of the AMOC is currently about 16°C at 26.5° N, so a 1°C change in this temperature difference would be equivalent to an AMOC change of about 1 Sv, in terms of their respective impacts on the total heat transport. Thus, an increase in upper ocean temperatures due to global warming would be expected to offset, to some degree, the anticipated reduction in the AMOC under global warming scenarios. It is also possible that the gyre's contribution to the heat transport will change as the world warms, and model studies have suggested that the gyre heat transport may increase to compensate for the reduction in overturning heat transport [95]. Since it is the variability of the MHT that is really of fundamental importance to climate, and to climate change, it is therefore more important to understand how the MHT will change rather than just the AMOC, which has tended to be the primary focus of IPCC model projections to date.

Finally, as shown in §3c, while it is possible to separate the contributions to the total heat transport into a zonally averaged vertical cell and a residual horizontal circulation cell, it is far from clear whether these can be taken as meaningful physical measures of the heat transport of the large-scale AMOC versus that of the gyre circulation. The topic has been addressed by several authors including Talley [72] based on observations, Xu *et al.* [78] based on numerical model results, and by Ferrari & Ferreira [77] and Yang *et al.* [96] based on the concept of a ‘heatfunction' that measures the overturning in temperature space rather than in depth or density space (see Xu *et al.* [78] for a particularly illuminating discussion of these different approaches). However, all of these approaches have in common that they require subjective decisions to be made on how to separate the shallow overturning circulation related to the wind-driven gyre circulation from that of the large-scale AMOC. It seems clear that further progress on this topic will require a Lagrangian-based approach in which water parcels in the upper limb of the AMOC are tracked as they move northward across a certain latitude, and their temperature changes are monitored until they re-cross that latitude either in the lower limb of the AMOC (i.e. below the maximum of the AMOC streamfunction) or within the upper limb of the AMOC. A similar approach has been used to track density changes along three-dimensional flow pathways in the subpolar gyre [97] but could be equally well applied to respective temperature changes and heat transport. The heat transport associated with water parcels that return in the lower limb of the AMOC would thus represent the heat transport of the large-scale shallow-to-deep AMOC cell, while the heat transport of those parcels staying in the upper limb of the cell would represent the heat transport by the gyre and shallow overturning circulation. The computational burden of integrating trajectories over very long time periods (e.g. for waters returning within the deep limb of the AMOC) could possibly be reduced by taking advantage of so-called ‘transit-matrix' approaches [98]. Such Lagrangian-based calculations might also help to shed light on how one would most accurately estimate these respective large-scale overturning and gyre heat transports from Eulerian-based observations such as the RAPID (and other) AMOC arrays in the Atlantic.

## 5. Summary

The RAPID-MOCHA-WBTS observing system along 26.5° N has been continuously operating for nearly two decades, with the currently available measurements of the AMOC and associated MHT now spanning the period from April 2004 to December 2020. The 26.5° N observations provide a valuable benchmark for testing ocean circulation models, coupled climate models and indirect methods used for estimating ocean heat transport via residual or surface flux methods. The main conclusions from this study are:

— The mean AMOC strength and MHT derived from the 2004 to 2020 record acquired thus far are 16.9 ± 1.2 Sv and 1.20 ± 0.12 PW, respectively. The observed MHT of 1.2 PW is consistent with the range of estimates from trans-basin hydrographic sections along 24–26° N but is larger than most estimates produced by coupled climate models, or from surface heat flux datasets derived from direct or indirect methods and from atmospheric models forced by observed surface boundary conditions.
— On interannual time scales the AMOC and MHT show changes of up to 20% with respect to their time-mean values, with the largest short-term reduction occurring in 2009–2010 and a longer (decadal-scale) reduction since 2009. These changes have been shown to have important impacts on ocean circulation and patterns of ocean heat content change over the North Atlantic.
— The overturning circulation accounts for more than 90% of the total MHT at 26.5° N when cast in terms of a zonally averaged vertical overturning cell, as it is traditionally estimated. However, a more physically meaningful assessment of the contributions by the large-scale AMOC versus the gyre and its related shallow wind-driven overturning circulation requires more information than is provided by Eulerian-based arrays such as RAPID, and Lagrangian techniques could possibly help to provide better estimates of these contributions.
— The relationship of the MHT to the AMOC has shown some variation over the RAPID period, with the MHT/AMOC ratio having shown an increase in recent years (since approx. 2016) that is associated primarily with a warming of the Florida Current. The question of how the MHT will change in the future, even in the face of a declining AMOC, and by how much, is a topic worthy of further exploration in climate change models.

A further urgent challenge in AMOC research is to better understand the roles of wind forcing versus surface buoyancy forcing in driving the observed AMOC changes at 26.5° N, as well as at other locations in the Atlantic where AMOC observing arrays are acquiring increasingly long records. In particular, the role of deep density variations, such as those that have occurred in the DWBC at 26.5° N and which have resulted in a significant decline in LNADW transport—and possibly contributed to the overall AMOC reduction since 2009—are important to better understand from a causal and dynamic perspective. More model studies are needed to diagnose the causes of the observed AMOC changes over the full length of the RAPID record, and to understand the sources and propagation of density anomalies from high-latitude regions to the subtropics and the role that they may play in the spreading of buoyancy-forced AMOC changes through the Atlantic.

## Data Availability

Original data and processed data products used in this study are publicly available at the following websites: https://rapid.ac.uk/rapidmoc/rapid_data/datadl.php; https://www.bodc.ac.uk/resources/inventories/edmed/report/6769/; https://mocha.earth.miami.edu/mocha/index.html; https://www.aoml.noaa.gov/phod/wbts/. The data can also be accessed at the following repositories: AMOC: doi:10.5285/e91b10af-6f0a-7fa7-e053-6c86abc05a09. MHT: https://doi.org/10.17604/3nfq-va20.
